# Supplementary material for: Detailed molecular and epigenetic characterization of the pig IPEC-J2 and chicken SL-29 cell lines
Source: iScience. 2023 Feb 20;26(3):106252. doi: 10.1016/j.isci.2023.106252 (PMC10018572; doi:10.1016/j.isci.2023.106252)
Supplement: Data S2. Complete homer output for identified motifs in Chicken SL-29, related to Tables 5 and 6 — Homer motif analysis results for histone modifications H3K4me1, H3K4me3, H3K27ac, enhancers, and ATAC-seq of chicken SL-29 cell line. Parameters for possible false positives is as mentioned earlier for S5. [file mmc3.zip › Data_S2/S6/Chicken_SL_29/peak_files_CTCF/homerResults/motif25.similar.html]

motif25

## Information for motif25

C
A
G
T
G
C
A
T
C
G
T
A
A
T
C
G
C
A
T
G
A
T
C
G
G
C
A
T
C
G
A
T
C
T
G
A
C
T
A
G
C
T
A
G
C
A
T
G
C
G
A
T
  
Reverse Opposite:  

G
C
T
A
G
T
A
C
G
A
T
C
G
A
T
C
G
A
C
T
G
C
T
A
C
G
T
A
T
A
G
C
G
T
A
C
T
A
G
C
G
C
A
T
C
G
T
A
G
T
C
A
  

|  |  |
| --- | --- |
| p-value: | 1e-10 |
| log p-value: | -2.482e+01 |
| Information Content per bp: | 1.695 |
| Number of Target Sequences with motif | 38.0 |
| Percentage of Target Sequences with motif | 0.13% |
| Number of Background Sequences with motif | 10.2 |
| Percentage of Background Sequences with motif | 0.04% |
| Average Position of motif in Targets | 154.0 +/- 83.2bp |
| Average Position of motif in Background | 149.4 +/- 47.9bp |
| Strand Bias (log2 ratio + to - strand density) | 2.3 |
| Multiplicity (# of sites on avg that occur together) | 7.34 |
| Motif File: | file (matrix) reverse opposite |

### Similar de novo motifs found

|  |  |  |  |  |  |  |  |
| --- | --- | --- | --- | --- | --- | --- | --- |
| Rank | Match Score | Redundant Motif | P-value | log P-value | % of Targets | % of Background | Motif file |
| 1 | 0.890 | T G A C G A C T G C T A G C T A T A G C T G A C T A G C C G A T C G T A G T C A | 1e-10 | -23.904525 | 0.17% | 0.06% | motif file (matrix) |
| 2 | 0.819 | C G T A C A T G C A T G A T C G C G A T G A C T C G T A C T A G C A T G C A T G C G A T G C A T C T G A | 1e-9 | -20.823734 | 0.24% | 0.11% | motif file (matrix) |
| 3 | 0.874 | T G A C T A G C C G A T C G T A G C T A A G T C G T A C T A G C C G A T C T G A | 1e-7 | -16.154278 | 0.24% | 0.12% | motif file (matrix) |
| 4 | 0.795 | A C G T C G T A C G T A A G T C A G T C A T G C A C G T C G T A | 1e-6 | -16.008155 | 0.47% | 0.30% | motif file (matrix) |
